# Hepatic Lipofuscin Deposition in Living Liver Donor Candidate

**DOI:** 10.7759/cureus.95770

**Published:** 2025-10-30

**Authors:** Takahiko Omameuda, Yasuharu Onishi, Yukihiro Sanada, Yasunaru Sakuma, Hironori Yamaguchi

**Affiliations:** 1 Division of Gastroenterological, General and Transplant Surgery, Department of Surgery, Jichi Medical University, Shimotsuke, JPN

**Keywords:** lipofuscin, liver biopsy, living liver donor, preoperative evaluation, transplantation

## Abstract

Living donor liver transplantation (LDLT) is an important therapeutic option, and ensuring donor safety is paramount. We report a case of a woman in her early 50s evaluated as a living liver donor candidate for her husband. Recurrent mild elevations of alanine aminotransferase (ALT) levels prompted further investigation, and a percutaneous ultrasound-guided liver biopsy revealed unexpected and marked lipofuscin deposition within hepatocytes, without evidence of steatosis, inflammation, fibrosis, or cholestasis. Lipofuscin, an age-related pigment, is generally considered benign, with an unclear association with hepatic dysfunction. In this case, the donor was excluded due to recurrent unexplained liver dysfunction rather than the presence of lipofuscin deposition itself. Histopathological evaluation plays a critical role in living donor selection, particularly when liver dysfunction is suspected. Furthermore, unexpected histopathological findings, such as lipofuscin deposition, serve to reaffirm the necessity of thorough interpretation and comprehensive discussion within the transplant team, with careful consideration of donor safety and graft functionality.

## Introduction

Living donor liver transplantation (LDLT) has long been an established therapeutic option for end-stage liver disease, particularly in Japan, where deceased donor organs have consistently been in short supply [1]. The safety of the living donor is paramount [2], and careful selection based on clinical, biochemical, radiologic, and histologic parameters is therefore critical. Although various biochemical, anthropometric, and radiologic methods have been extensively evaluated, percutaneous liver biopsy, despite its invasiveness, remains the criterion standard for determining graft pathology [3].

At our institution, when liver dysfunction is identified in potential living liver donors, a percutaneous liver biopsy is performed after obtaining informed consent to determine the underlying cause. In general, macrosteatosis and total steatosis are widely recognized as factors that may affect graft function [4]. Macrosteatosis, defined as the excessive accumulation of fat within hepatocytes that can reduce graft viability and compromise postoperative outcomes, underscores the importance of histopathological evaluation by liver biopsy for assessing graft function while also potentially playing a critical role in evaluating donor safety.

We performed a liver biopsy in a living donor candidate with recurrent liver dysfunction and unexpectedly observed excessive lipofuscin deposition in the liver. Lipofuscin is an intralysosomal, autofluorescent pigment that accumulates over time due to oxidative stress and aging [5-7]. It is often called age pigment [8]. Lipofuscin consists of light brown granules and is found in tissues such as the heart, liver, kidney, neuronal tissue, skeletal muscle cells, and dermis [9-12]. In the aging liver, it contributes to morphological changes, including darkening due to its accumulation [13]. Currently, there is no available treatment to remove it. Accurate large-scale epidemiological data on the frequency of hepatic lipofuscin deposition are lacking, and its clinical significance remains largely unclear. Although a previous report has suggested a potential association between chronic liver disease and lipofuscin accumulation [14], previous studies have not provided definitive evidence that lipofuscin deposition in donor livers compromises donor safety, and it is generally considered to have no significant impact on graft function. Ultimately, although the donor candidate was deemed ineligible due to unexplained liver dysfunction, the histopathological evaluation from the liver biopsy served to reaffirm to the transplant team the critical importance of carefully considering both donor safety and the potential impact on graft function.

## Case presentation

A woman in her early 50s was evaluated as a potential living liver donor for her husband, who suffered from end-stage liver disease. Her medical history was unremarkable, with no known hepatic disorders, alcohol use, or metabolic syndrome. Her vital signs at presentation were within normal limits. Initial laboratory testing revealed a mild elevation of alanine aminotransferase (ALT) at 41 IU/L (Table 1), which subsequently decreased to 23 IU/L and normalized within approximately three months without any intervention (Table 2).

**Table 1:** Summary of the laboratory findings upon presentation ALP: alkaline phosphatase; ALT: alanine aminotransferase; AMY: amylase; ANA: anti-nuclear antibody; AST: aspartate aminotransferase; AFP: alpha-fetoprotein; Alb: albumin; APTT: activated partial thromboplastin time; BUN: blood urea nitrogen; activated partial thromboplastin time; CA125: cancer antigen 125; CA19-9: carbohydrate antigen 19-9; CEA: carcinoembryonic antigen; ChE: cholinesterase; Cre: creatinine; CRP: C-reactive protein; D-bil: direct bilirubin; GGT: γ-gutamyl transpeptidase; Hb: hemoglobin; HBs-Ag: hepatitis B surface-antigen; HCV-Ab: hepatitis C virus-antibody; Ht: hematocrit; IgG: immunoglobulin G; LDH: lactate dehydrogenase; Plt: platelet count; PT-INR:  prothrombin time-international normalized ratio;  PIVKA-II: protein induced by vitamin K absence-II; RBC: red blood cells; TBA: total bile acid,  T-bil: total bilirubin; TP: total protein; WBC: white blood cells

Parameter	Result (Reference Range)	Parameter	Result (Reference Range)
Peripheral blood		AST	27 U/L (13-30 U/L)
WBC	5.7 x10^3 /µL (3.3-8.6x10^3 /µL)	ALT	41 U/L (7-23 U/L)
RBG	4.81 x10^6/µL (3.86-4.92x10^6 /µL)	LDH	206 U/L (124-222 U/L)
Hb	14.2 g/dL (11.6-14.8 g/dL)	GGT	16 U/L (9-32 U/L)
Ht	43.2 % (35.1-44.4 %)	TBA	2.7 µmol/L (<12.0 µmol/L)
Plt	241 x10^3 /µL (158-348 x10^3 /µL)	ALP	77 U/L (38-113 U/L)
Coagulation		ChE	204 U/L (201-421 U/L)
PT-INR	0.97 (0.85-1.15)	AMY	75 U/L (44-132 U/L)
APTT	29.1 sec (22.4-37.4 sec)	Ferritin	34.7 ng/mL (5-152 ng/mL)
Biochemistry		ANA	- (-)
CRP	0.02 mg/dL (0.00-0.14 mg/dL)	IgG	1330 mg/dL (861-1747 mg/dL)
TP	7.4 g/dL (6.6-8.1 g/dL)	HBs-Ag	0.00 IU/m (- IU/mL)
Alb	4.6 g/dL (4.1-5.1 g/dL)	HCV-Ab	0.07 S/CO (- S/CO)
BUN	13 mg/dL (8-20 mg/dL)	Tumor markers	
Cre	0.65 mg/dL (0.46-0.79 mg/dL)	CEA	3.9 ng/mL (<4.5 ng/mL)
T-bil	0.79 mg/dL (0.40-1.50 mg/dL)	CA19-9	<1 U/mL (<36 U/mL)
D-bil	0.06 mg/dL (0.06-0.23 mg/dL)	CA125	11 U/mL (<35 U/mL)

**Table 2:** Summary of the laboratory findings upon clinical improvement WBC: White blood cells, RBG: Random blood glucose, Hb: hemoglobin, Plt: platelet count, CRP: C-reactive protein, TP: total protein, Alb: albumin, BUN: blood urea nitrogen

Parameter	Result (Reference Range)	Parameter	Result (Reference Range)
Peripheral blood		Cre	0.71 mg/dL (0.46-0.79 mg/dL)
WBC	4.9 x10^3 /µL (3.3-8.6x10^3 /µL)	T-bil	0.57 mg/dL (0.40-1.50 mg/dL)
RBG	4.27 x10^6/µL (3.86-4.92x10^6 /µL)	D-bil	0.03 mg/dL (0.06-0.23 mg/dL)
Hb	12.4 g/dL (11.6-14.8 g/dL)	AST	20 U/L (13-30 U/L)
Plt	200 x10^3 /µL (158-348 x10^3 /µL)	ALT	23 U/L (7-23 U/L)
Biochemistry		LDH	185 U/L (124-222 U/L)
CRP	0.01 mg/dL (0.00-0.14 mg/dL)	GGT	13 U/L (9-32 U/L)
TP	6.6 g/dL (6.6-8.1 g/dL)	TBA	4.5 µmol/L (<12.0 µmol/L)
Alb	4.1 g/dL (4.1-5.1 g/dL)	ALP	71 U/L (38-113 U/L)
BUN	13 mg/dL (8-20 mg/dL)	ChE	175 U/L (201-421 U/L)

**Figure 1:**
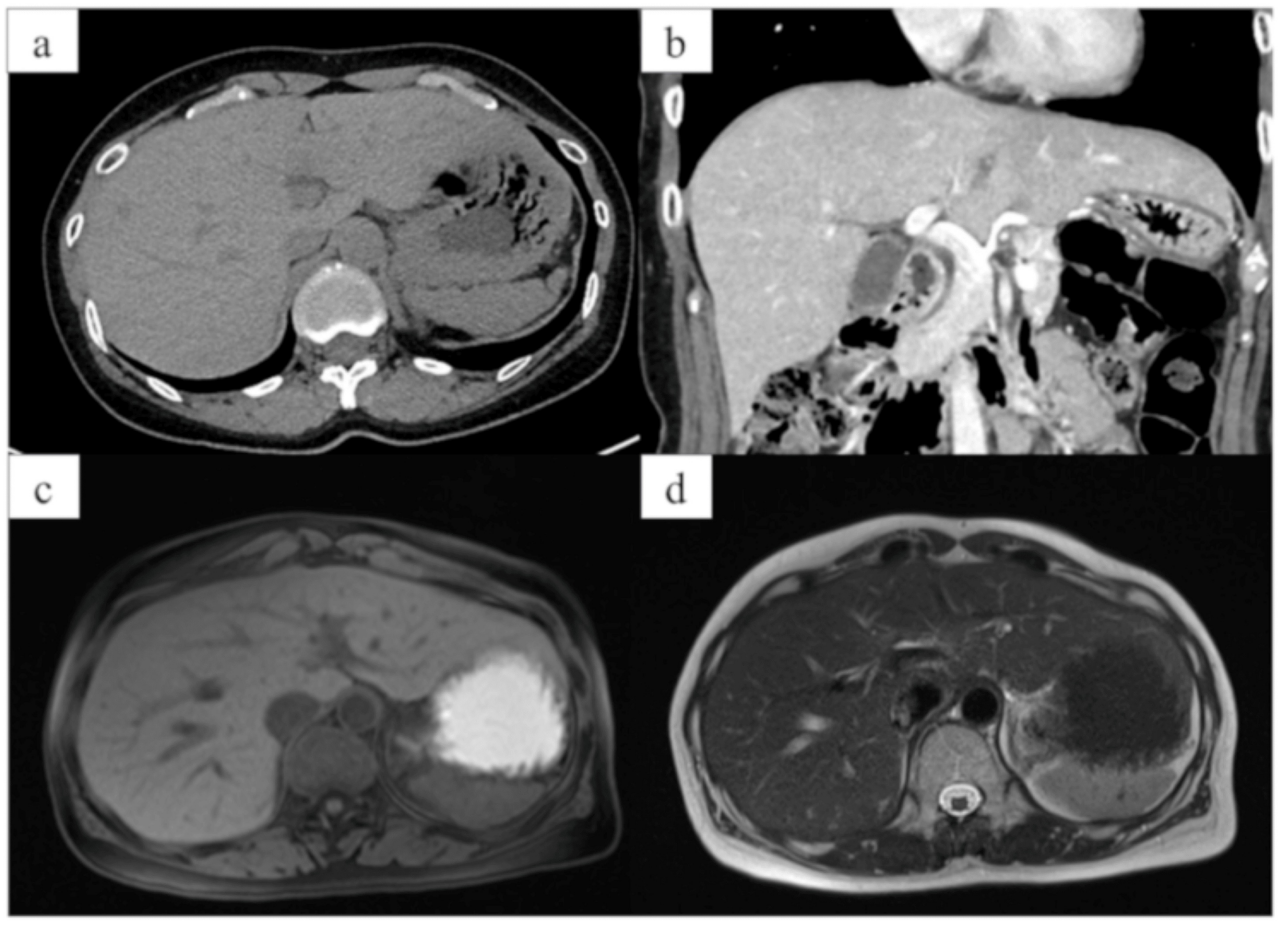
Imaging of a living liver donor candidate a: Unenhanced computed tomography (CT) showed no evidence of hepatic steatosis. b: Contrast-enhanced CT revealed no remarkable abnormalities. c: No significant abnormalities were observed on T1-weighted magnetic resonance imaging (MRI). d: No significant abnormalities were observed on T2-weighted magnetic MRI. CT and MRI showed no evidence of hepatic steatosis or structural abnormalities.

Imaging included computed tomography (Figures 1a, 1b) and magnetic resonance imaging (Figures 1c, 1d), which showed no evidence of hepatic steatosis or structural abnormalities. She was initially deemed eligible for donation. However, approximately one month after normalization of liver enzyme levels and prior to the scheduled surgery, repeat liver function tests revealed an ALT level of 43 IU/L (Table 3).

**Table 3:** Summary of the laboratory findings prior to surgery WBC: White blood cells, RBG: Random blood glucose, Hb: hemoglobin, Plt: platelet count, CRP: C-reactive protein, TP: total protein, Alb: albumin, BUN: blood urea nitrogen

Parameter	Result (Reference Range)	Parameter	Result (Reference Range)
Peripheral blood		Cre	0.64 mg/dL (0.46-0.79 mg/dL)
WBC	6.1 x10^3 /µL (3.3-8.6x10^3 /µL)	T-bil	0.68 mg/dL (0.40-1.50 mg/dL)
RBG	4.78 x10^6/µL (3.86-4.92x10^6 /µL)	D-bil	0.01 mg/dL (0.06-0.23 mg/dL)
Hb	13.6 g/dL (11.6-14.8 g/dL)	AST	29 U/L (13-30 U/L)
Plt	227 x10^3 /µL (158-348 x10^3 /µL)	ALT	43 U/L (7-23 U/L)
Biochemistry		LDH	198 U/L (124-222 U/L)
CRP	0.03 mg/dL (0.00-0.14 mg/dL)	GGT	20 U/L (9-32 U/L)
TP	7.0 g/dL (6.6-8.1 g/dL)	TBA	4.2 µmol/L (<12.0 µmol/L)
Alb	4.2 g/dL (4.1-5.1 g/dL)	ALP	85 U/L (38-113 U/L)
BUN	14 mg/dL (8-20 mg/dL)	ChE	192 U/L (201-421 U/L)

Although the elevation was mild, the recurrence prompted further evaluation to ensure the safety of the potential donor. A percutaneous ultrasound-guided liver biopsy was performed to investigate the cause of recurrent elevations in liver enzymes.

**Figure 2:**
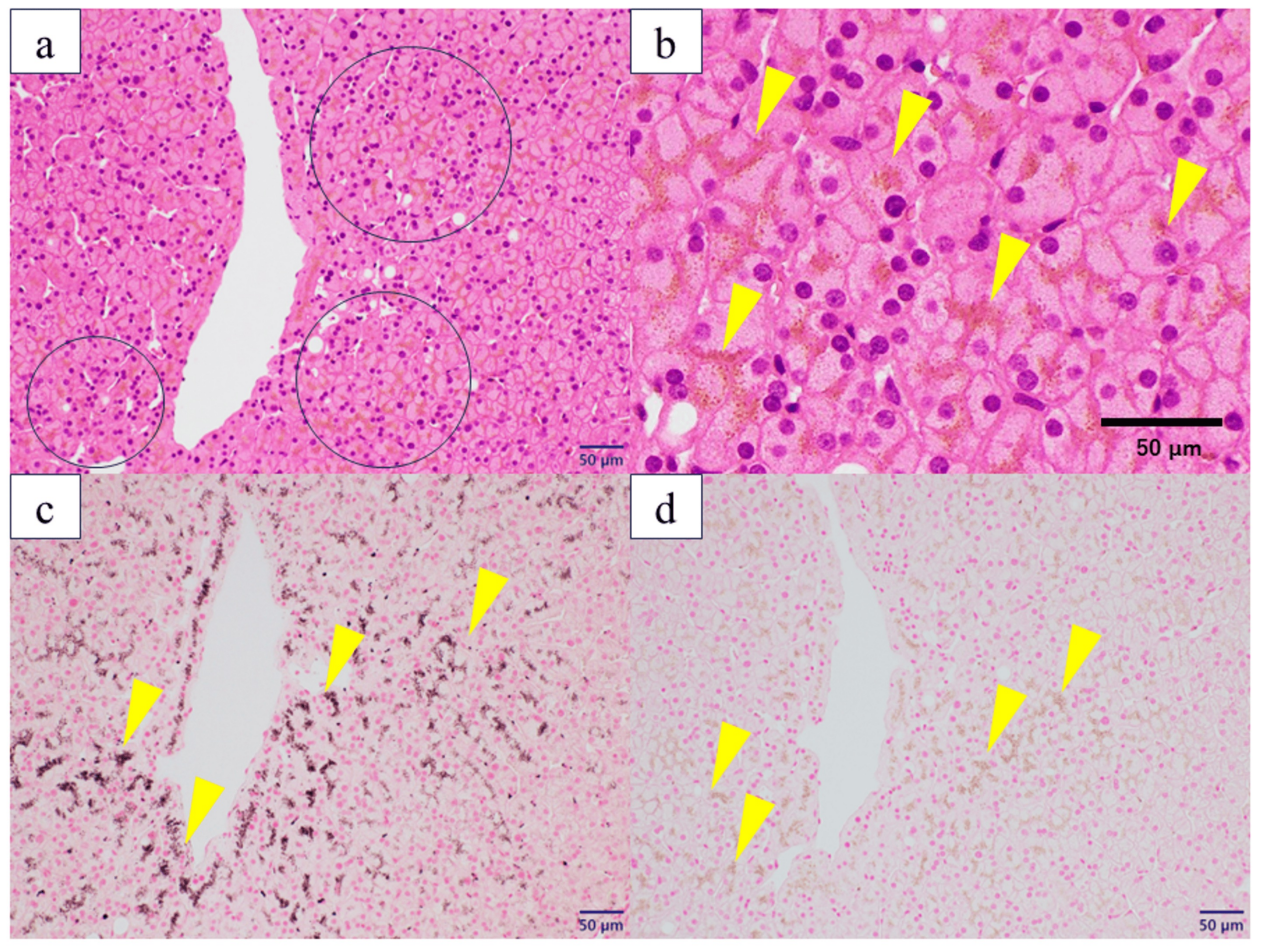
Pathological findings of a living liver donor candidate a: Histopathological examination with hematoxylin and eosin (H＆E) staining revealed numerous hepatocytes containing light brown granules. Open circles indicate hepatocytes containing light brown granules (H＆E staining, X100）. b: A magnified view of (a). Arrowheads indicate hepatocytes containing light brown granules, which correspond to lipofuscin. c: Granules which were positive for Fontana-Masson staining. Arrowheads highlight Fontana–Masson–positive granules (Fontana-Masson staining, X200). d: Granules which were negative for iron staining. Arrowheads highlight iron staining –negative granules. Negative iron stain excludes hemosiderin (iron staining, X200). No evidence of steatosis, inflammation, fibrosis, or cholestasis was observed. Based on these findings, the granules were diagnosed as excessive deposition of lipofuscin within hepatocytes. Bars indicate 50 μm in 2a, b, c, and d.

Histological examination with hematoxylin and eosin staining revealed fine, light-brown granules within the cytoplasm of hepatocytes (Figures 2a, 2b). These granules were positive on Fontana-Masson staining (Figure 2c) and negative on iron staining (Figure 2d).

No evidence of steatosis, inflammation, fibrosis, or cholestasis was observed. Based on these findings, the granules were ultimately diagnosed as excessive deposition of lipofuscin within hepatocytes.

The candidate was ultimately deemed unsuitable as a living donor due to recurrent liver dysfunction; outpatient follow-up with periodic blood tests was planned to monitor liver function. However, the unexpected identification of lipofuscin deposition on liver biopsy prompted reconsideration of donor eligibility.

## Discussion

This case highlighted two important clinical implications. First, liver biopsy serves as a valuable diagnostic tool not only for the evaluation of hepatic steatosis but also for identifying other potential hepatic abnormalities. Second, when incidental histopathological findings are encountered through liver biopsy, it is essential to re-evaluate donor eligibility from the perspectives of both graft function and donor safety.

In LDLT, donor safety is the top priority. However, it is not uncommon for individuals initially considered as potential donors to present with abnormal liver function. Although eligibility criteria for living donors vary among institutions, our policy is to perform a liver biopsy to investigate the underlying cause of liver dysfunction after obtaining informed consent and providing a thorough explanation, particularly in cases where an alternative donor is not available. In living donor evaluations, liver biopsy is typically performed to assess hepatic steatosis, as ≥10% hepatic steatosis often precludes donation due to concerns regarding donor safety and recipient outcomes [15]. In the present case, although steatosis was absent, excessive lipofuscin deposition within hepatocytes was identified. Lipofuscin is an intralysosomal, autofluorescent pigment that accumulates over time due to oxidative stress and aging [5-7], and several steps of lipofuscinogenesis are not yet fully understood. It is often called age pigment and regarded as a hallmark of aging, not only because it increases with age but also due to its nearly linear accumulation over time [8]. Although a direct association between lipofuscin deposition and hepatic dysfunction remains unclear, in the present case, a liver biopsy performed to investigate elevated liver enzymes incidentally revealed excessive lipofuscin deposition. The underlying cause of hepatic dysfunction, however, remained unidentified. While the lipofuscin deposition was an unexpected histopathological finding, it could not have been detected by imaging studies alone. Therefore, pathological assessment through liver biopsy was considered valuable in this context.

In the context of living liver donation, donor eligibility must be carefully assessed by balancing both donor safety and the potential impact on graft function. As demonstrated in this case, even incidental findings such as marked lipofuscin deposition should prompt a thorough evaluation from both perspectives. Previous reports have not demonstrated any definitive evidence that lipofuscin deposition in donor livers compromises donor safety; however, several studies and discussions have addressed its potential implications for graft function. A donor-liver study conducted in 1988 involving 30 cases reported an association between lipofuscin accumulation in donor livers and poor graft outcomes [16]. However, a subsequent study in 2009 analyzing liver biopsy specimens from 294 donor candidates found no significant correlation between lipofuscin deposition and the graft outcomes [4]. In addition, Melin C et al. have listed lipofuscin accumulation among the morphologic features that are not contraindications to transplantation [17]. Although the clinical significance of lipofuscin deposition in transplanted livers remains incompletely defined, at present, lipofuscin deposition in the graft liver alone is not considered a contraindication for donor eligibility. As this report pertains to a single case, opportunities for quantitative analysis and clinical correlation are inherently limited. However, in the present case, the donor was ultimately deemed ineligible due to recurrent episodes of unexplained liver dysfunction, rather than the presence of lipofuscin itself. Although there are no universally established cutoff values for liver function tests, persistently abnormal results or evidence of significant hepatic dysfunction are generally considered reasons to defer donation. Furthermore, given the increasing age of living donors in recent years, the incidence of lipofuscin accumulation is expected to rise in the future.

## Conclusions

This case highlights the critical role of histopathological assessment in evaluating living liver donor candidates with suspected liver dysfunction. Although lipofuscin deposition is generally regarded as a marker of cellular aging rather than a pathological entity, its incidental identification through liver biopsy prompted a careful reconsideration of donor eligibility. While the definitive cause of hepatic dysfunction remained unclear, this case underscored the value of liver biopsy not only in evaluating hepatic steatosis but also in revealing unexpected pathological changes. The observed data demonstrated incidental lipofuscin accumulation, and importantly, for such incidental histological findings, their clinical implications, regarding both donor safety and potential graft function, should be thoroughly interpreted and discussed within the transplant team.

## References

[1] Shimada M, Fujii M, Morine Y (2005). Living-donor liver transplantation: present status and future perspective. J Med Invest.

[2] Umeshita K, Fujiwara K, Kiyosawa K (2003). Operative morbidity of living liver donors in Japan. Lancet.

[3] Brandhagen D, Fidler J, Rosen C (2003). Evaluation of the donor liver for living donor liver transplantation. Liver Transpl.

[4] Fiorentino M, Vasuri F, Ravaioli M (2009). Predictive value of frozen-section analysis in the histological assessment of steatosis before liver transplantation. Liver Transpl.

[5] Brunk UT, Terman A (2002). Lipofuscin: mechanisms of age-related accumulation and influence on cell function. Free Radic Biol Med.

[6] Brunk UT, Jones CB, Sohal RS (1992). A novel hypothesis of lipofuscinogenesis and cellular aging based on interactions between oxidative stress and autophagocytosis. Mutat Res.

[7] Cindrova-Davies T, Fogarty NM, Jones CJ (2018). Evidence of oxidative stress-induced senescence in mature, post-mature and pathological human placentas. Placenta.

[8] Jung T, Bader N, Grune T (2007). Lipofuscin: formation, distribution, and metabolic consequences. Ann N Y Acad Sci.

[9] ST BL, MA DD, MI AS, GEE MV (1959). Rate and magnitude of age pigment accumulation in the human myocardium. J Gerontol.

[10] Beregi E, Regius O, Hüttl T, Göbl Z (1988). Age-related changes in the skeletal muscle cells. Z Gerontol.

[11] Brunk U, Ericsson JL (1972). Electron microscopical studies on rat brain neurons. Localization of acid phosphatase and mode of formation of lipofuscin bodies. J Ultrastruct Res.

[12] Del Roso A, De Tata V, Gori Z, Bergamini E (1991). Transmural differences of lipofuscin pigment accumulation in the left ventricule of rat heart during growth and aging. Aging (Milano).

[13] Chatterjee N, Sharma R, Kale PR (2024). Is the liver resilient to the process of ageing?. Ann Hepatol.

[14] Saif M, Kwanten WJ, Carr JA (2020). Non-invasive monitoring of chronic liver disease via near-infrared and shortwave-infrared imaging of endogenous lipofuscin. Nat Biomed Eng.

[15] Hickey RD, Mao SA, Amiot B (2015). Noninvasive 3-dimensional imaging of liver regeneration in a mouse model of hereditary tyrosinemia type 1 using the sodium iodide symporter gene. Liver Transpl.

[16] Lanir A, Jenkins RL, Caldwell C (1988). Hepatic transplantation survival: correlation with adenine nucleotide level in donor liver. Hepatology.

[17] Melin C, Miick R, Young NA (2013). Approach to intraoperative consultation for donor liver biopsies. Arch Pathol Lab Med.

